# Air evolution during drop impact on liquid pool

**DOI:** 10.1038/s41598-020-62705-5

**Published:** 2020-04-01

**Authors:** Ji San Lee, Byung Mook Weon, Su Ji Park, Ji Tae Kim, Jaeyeon Pyo, Kamel Fezzaa, Jung Ho Je

**Affiliations:** 1 X-ray Imaging Center, Department of Materials Science and Engineering, Pohang University of Science and Technology, 77 Cheongam-Ro, Nam-Gu, Pohang, 37673 South Korea; 2 Soft Matter Physics Laboratory, School of Advanced Materials Science and Engineering, SKKU Advanced Institute of Nanotechnology (SAINT), Sungkyunkwan University, Suwon, 16419 South Korea; 3 Research Center for Advanced Materials Technology, Sungkyunkwan University, Suwon, 16419 South Korea; 4 Department of Biomedical Engineering, Johns Hopkins University, Baltimore, Maryland 21218 USA; 5 X-ray Science Division, Advanced Photon Source, Argonne National Laboratory, 9700 South Cass Avenue, Argonne, Illinois 60439 USA

**Keywords:** Fluid dynamics, Soft materials

## Abstract

We elucidate the evolution of the entrained air in drop impact on a wide range of liquids, using ultrafast X-ray phase-contrast imaging. We elaborate the retraction mechanism of the entrapped air film in terms of liquid viscosity. We found the criterion for deciding if the entrapped air evolves into single or double bubbles, as determined by competition among inertia, capillarity, and viscosity. Low viscosity and low surface tension induce a small daughter droplet encapsulated by a larger air shell bubble, forming an antibubble. We demonstrate a phase diagram for air evolution regarding hydrodynamics.

## Introduction

Drop impact on a liquid surface has great importance in many natural and industrial processes. Raindrops or breaking waves can entrain small air bubbles when they fall onto the sea. This process is a crucial mechanism of gas transport from the atmosphere to the ocean and plays a crucial role in climate change and the ecosystem^1–4^, and also of great interest in fundamental science and technology. For example, entrapped bubbles can produce underwater noises by their oscillation or enhance nucleate boiling in chemical processes^5–7^. On solid substrates, air entrapment in drop impact has been actively studied^8–14^. The air underneath an impacting drop fails to drain and is instead compressed, deforming the bottom surface of the drop. The air layer ruptures thereby, resulting in the entrapment of an air film, eventually evolving into formation of a bubble^11–14^. On liquid surfaces, however, the evolution of the air entrapment has been largely unexplored because of difficulty in visualizing its microscale (<100 *μ*m), rapid (<100 *μ*s), and complex dynamics^15–23^. In particular, there is currently no physical model that exactly predicts the morphology of the entrapped bubble in a wide range of liquid properties and impact conditions.

In this paper, we studied the evolution of the air entrained by drop impact on a variety of liquid pools by using high-speed X-ray phase-contrast imaging that enables us to clearly track the rapid evolution of the interfaces in high temporal (~*μ*s) and spatial (~*μ*s) resolutions. The retraction mechanism of an entrapped air film is elaborated in terms of liquid viscosity. The criterion for deciding if the entrapped air evolves into single or double bubbles is rationalized, based on competition among inertia, capillarity, and viscosity. Additionally, it was found that low viscosity and low surface tension induce formation of a small daughter droplet encapsulated by an air shell, resulting in formation of an antibubble. A complete phase diagram for air evolution is demonstrated with respect to hydrodynamic conditions.

## Experiments

We studied the impact of a liquid drop on a pool of the same liquid with X-ray imaging. To achieve high-intensity light source, white-beam X-ray with a peak irradiance of ~10^14^ ph/s/mm^2^/0.1% bw was used^24,25^. The detector system comprises a fast scintillator crystal (LuAG:Ce, decay time ~50 ns), a right-angle mirror and the microscope objective (Mitutoyo M Plan APO 10x, NA = 0.21). The images were captured with a CMOS high-speed camera (Photron Fastcam SA1.1). The imaging speed of the camera was synchronized to the X-ray beam using delay generators, enabling us to capture images with period of 3.68 *μ*s and exposure time of 472 ns^24,25^. The drop-impact setup was installed 150 mm distance from the detector to achieve a strong phase-contrast effect. Liquid drops were dispensed from a syringe needle (26 G) connected with a remote-controlled syringe pump, resulting the droplet diameter to be 1.9 ~ 2.6 mm for different liquids. A laser beam was used to sense the drop and trigger the camera and the fast shutter that is installed before the sample stage. The liquid pool for the substrate was prepared in a cylinder made by Kapton film with a diameter ~20 mm and a depth ~50 mm, considered as a sufficiently deep pool^19^. The ambient temperature was carefully controlled to 20 °C in every experiment.

## Results

### X-ray imaging experiments

**Figure 1:**
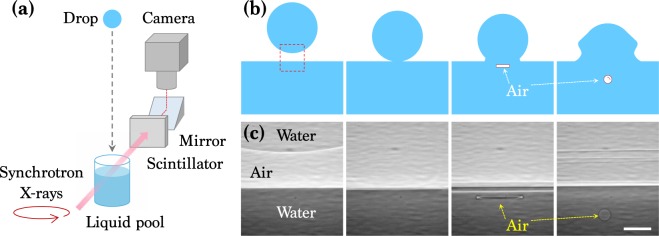
X-ray imaging setup and air bubble entrapment by drop impact on a liquid pool. (**a**) Schematic of X-ray imaging coupled with drop-impact setup. (**b**) Schematic of the bubble entrainment. When a liquid drop falls onto a liquid pool, a thin air film is entrapped and eventually contracts to one bubble or a few bubbles by surface energy minimization. (**c**) X-ray images corresponding to sketches in (**b**). The scale bar is 100 *μ*m long.

Wo used X-ray imaging experiments equipped with a drop-impact setup, as illustrated in Fig. 1(a). The experiments were conducted at XSD 32-ID undulator beamline of the Advanced Photon Source in the Argonne National Laboratory. The air entrainment during drop impact on a liquid pool, as depicted in Fig. 1(b), was successfully taken as shown in Fig. 1(c). The imaging speed of the camera was synchronized to the X-ray beam using delay generators, enabling us to capture images with period of 3.68 *μ*s and exposure time of 472 ns^24–26^. To investigate the effects of liquid viscosity (*μ*) and surface tension (*γ*), we tested two model systems: i) alkane oils (heptane (C_7_H_16_), dodecane (C_12_H_26_), and pentadecane (C_15_H_32_)) and ii) mixtures of water and glycerol (W_*x*_-G_*y*_) where *x* and *y* denote mass fractions; 0, 0.2, 0.4, 0.6, 0.8, and 1.0. The liquid properties at temperature *T* = 293 K are summarized in Table 1, where all data were retrieved from the literature^27–34^. The diameters of liquid drops (*D*) were ~1.9 mm for oils and ~2.6 mm for W-G. The drop releasing heights (*H*) were controlled as 8 ~ 30 cm, corresponding to the impact velocities (*U*) as 1.25 ~ 2.4 m/s. The Weber number for the impacting drop was We_*drop*_ = *ρU*^2^*D*/*γ* = 50 ~ 210, where *ρ* is the liquid density, and the Froude number for the impacting drop was Fr_*drop*_ = *U*^2^/*gD* = 60 ~ 180, where *g* is the gravitational acceleration. Here the experimental conditions belong to an intermediate regime between bouncing and splashing^35^.

**Table 1:** Properties of liquids at *T* = 293 K. Here *x*_*G*_ refers water-glycerol mixtures with a fraction of glycerol. All data were retrieved from the literature^27– 34^.

Liquids	*ρ* (kg m^−3^)	*μ* (mPa s)	*γ* (mN m^−1^)
*x*_*G*_ = 0.0	998.0 (ref. ^27^)	1.005 (ref. ^27^)	72.0 (ref. ^28^)
*x*_*G*_ = 0.2	1051.2 (ref. ^27^)	1.742 (ref. ^27^)	69.5 (ref. ^28^)
*x*_*G*_ = 0.4	1100.4 (ref. ^27^)	3.685 (ref. ^27^)	67.9 (ref. ^28^)
*x*_*G*_ = 0.6	1157.6 (ref. ^27^)	10.911 (ref. ^27^)	66.9 (ref. ^28^)
*x*_*G*_ = 0.8	1210.7 (ref. ^27^)	60.856 (ref. ^27^)	65.7 (ref. ^28^)
*x*_*G*_ = 1.0	1263.9 (ref. ^27^)	1413.8 (ref. ^27^)	62.5 (ref. ^28^)
Heptane	683.59 (ref. ^29^)	0.415 (ref. ^30^)	21.5 (ref. ^31^)
Dodecane	746.4 (ref. ^32^)	1.500 (ref. ^30^)	25.6 (ref. ^33^)
Penrtadecane	768.3 (ref. ^34^)	2.841 (ref. ^34^)	27.12 (ref. ^33^)

**Figure 2:**
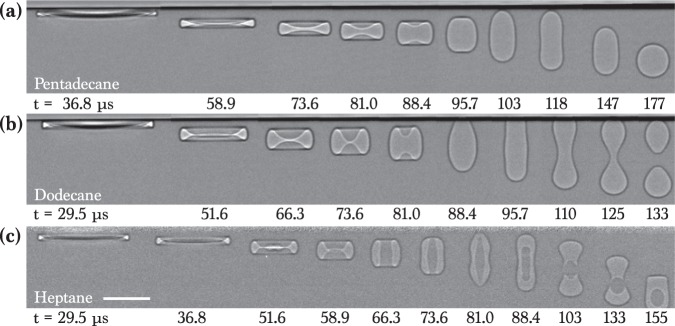
X-ray images of air evolution for oils. (**a**) Impact of the pentedecane drop (*μ* = 2.8 mPa s) at *H* = 8 cm, indicating one bubble. (**b**) Impact of the dodecane drop (*μ* = 1.5 mPa s) at *H* = 8 cm, indicating a double bubble. (**c**) Impact of the heptane drop (*μ* = 0.4 mPa s) at *H* = 16 cm, indicating an antibubble. All individual images were taken with one X-ray pulse of 472 ns long. All the images were background-corrected. The time at impact moment was set to *t* = 0. The scale bar is 100 *μ*m long.

Figure 2 representatively shows sequential X-ray images that demonstrate the evolution of the entrained air in drop impact on a liquid pool for alkane liquids with small *γ* (20 ~ 27 mN/m). The external shape as well as the exact internal morphology of the entrained air are clearly visualized in high spatial resolution (~2 *μ*m). The evolution from initial air films to final bubbles seems very elegant but quite complicated to understand. For the pentadecane in Fig. 2(a) and Movie S1, the air film initially evolves into a pancake-shape, is stretched into a vertical column at 118 *μ*s (note that this bubble does not touch the upper interface. The upper horizontal black line is because of the projection of the undisturbed front and back interfaces of the impacted region in X-ray penetration), and finally becomes one bubble at 177 *μ*s. For the dodecane in Fig. 2(b) and Movie S2 with a lower viscosity, a longer vertical air column at 96 ~ 110 *μ*s is formed and split into a double bubble (two bubbles) at 133 *μ*s. For the heptane in Fig. 2(c) and Movie S3 with a much lower viscosity, the evolution becomes more complicated as follow. The central region of a pancake-like air film is punctured during retraction, forming a toroidal shape at 59 ~ 66 *μ*s. The vertical stretching of the toroidal bubble at 66 ~ 81 *μ*s induces the pinch-off of a daughter droplet at 81 ~ 88 *μ*s, finally encapsulated by an air shell, forming an antibubble. The antibubble seems to have a similar configuration with a typical antibubble^36,37^, but the generation principle is quite different. Whereas the surfactant-stabilized antibubble is formed by oriented surfactant molecules that provide some elasticity to the air/liquid interfaces and ensure air-shell stability^36,37^, the origin of the the surfactant-free antibubble is the entrapped air by drop impact. These results clearly demonstrate that generation of single or double bubbles and antibubbles can be controlled during drop impact on a liquid pool by simply manipulating liquid viscosity.

**Figure 3:**
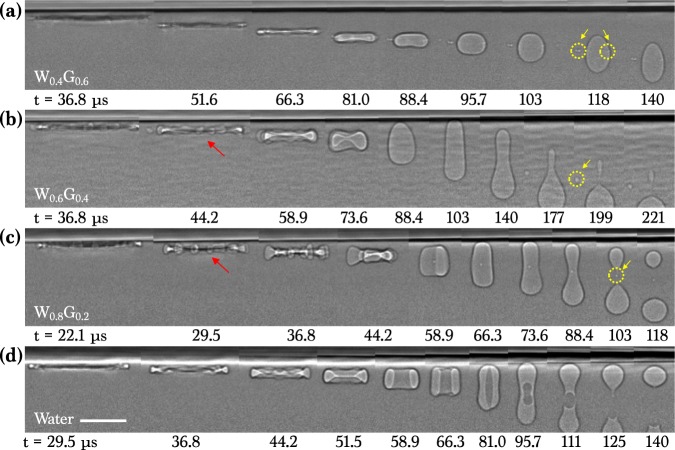
X-ray images of air evolution for water-glycerol mixtures (W-G). Imaging condition is the same with Fig. 2. (**a**) Impact of a W_0.4_G_0.6_ drop (*μ* = 10.9 mPa s) at *H* = 12 cm. (**b**) Impact of a W_0.6_G_0.4_ drop (*μ* = 3.7 mPa s) at *H* = 12 cm. (**c**) Impact of a W_0.8_G_0.2_ drop (*μ* = 1.7 mPa s) at *H* = 12 cm. (**d**) Impact of a water drop (*μ* = 1.0 mPa s) at *H* = 12 cm. Here, one bubble in (**a**), a double bubble in (**b–d**), and additional tiny bubbles (yellow circles) were generated. The scale bar is 100 *μ*m long.

For a water-glycerol mixture with large *γ* (62 ~ 72 mN/m), we observed a similar viscosity-dependent air evolution, as demonstrated in Fig. 3. During retraction, the air evolved into one bubble for the high-viscosity W_0.4_G_0.6_ as shown in Fig. 3(a) and Movie S4 but split into a double bubble (two bubbles) for the intermediate-viscosity W_0.6_G_0.4_ in Fig. 3(b) and Movie S5 as well as W_0.8_G_0.2_ in Fig. 3(c) and Movie S6. For the low-viscosity pure water, a daughter droplet was formed in Fig. 3(d) and Movie S7 but the toroidal bubble was eventually split at 96 ~ 111 *μ*s into a double bubble. Here we note that the initial retracting discs show undulation (red arrows in Fig. 3) and tiny bubbles were formed in the later stages (yellow circles in Fig. 3).

### Retraction dynamics

The retraction of the entrained air film in drop impact on a liquid pool has been studied mostly in inviscid liquids^17^. Here, we systematically investigated retraction dynamics in a wide range of liquids. The retraction dynamics of a thin fluid sheet is governed by the competition among inertia, capillarity, and viscosity^38–40^. Here, we describe the retraction of an air film. For simplicity, the air film is considered as a flat disc with a radius *R* and a thickness *δ*. For inviscid fluids, the retraction speed of the air disc determined by a balance between capillary and inertia can be approximated as *dR*/*dt* ≈ *C*_*i*_*R*/*τ*_*i*_, where *C*_*i*_ is the inertial proportional coefficient and *τ*_*i*_ is the inertial relaxation time^38,41^ as defined as ${\tau }_{i}=\sqrt{\rho (4/3)\pi {R}_{B}^{3}/\gamma }$ with *R*_*B*_ as the radius of the final spherical bubble. By a simple integration of the equation of retraction speed, we obtain an equation for inertial retraction^40^:

1 $$R(t)\approx {R}_{0}\ \exp (-{C}_{i}t/{\tau }_{i}),$$

where *R*_0_ is the initial radius of the disc. For viscous fluids, the retraction speed determined by a balance between capillary and viscosity can be approximated as *dR*/*dt* ≈ *C*_*v*_*R*/*τ*_*v*_, where *C*_*v*_ is the viscous proportional coefficient and *τ*_*v*_ is the viscous relaxation time^38,41–43^ as defined as *τ*_*v*_ = *μR*_*B*_/*γ*. Eventually, we obtain an equation for viscous retraction:

2 $$R(t)\approx {R}_{0}\ \exp (-{C}_{v}t/{\tau }_{v}).$$

The ratio of the two timescales, ${\tau }_{i}/{\tau }_{v}=\sqrt{(4/3)\rho \gamma {R}_{B}}/\mu$, corresponds to the inverse Ohnesorge number (Oh^−1^): Oh $=\mu /\sqrt{\rho \gamma {R}_{B}}$ represents the effect of viscosity over inertia and surface tension^13^.

**Figure 4:**
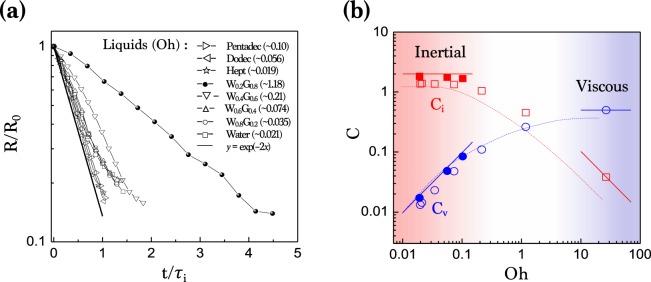
Retraction dynamics of the entrapped air film. (**a**) The film radius *R* measured with time *t* for oils and W-G mixtures. Here *R* and *t* are rescaled with *R*_0_ and *τ*_*i*_, respectively. The solid line is for the theoretical inertial retraction, $R(t)/{R}_{0}=\exp (-{C}_{i}t/{\tau }_{i})$, with *C*_*i*_ taken as 2^38,40,41^. (**b**) The best-fit values of the proportional coefficients of liquids, *C*_*i*_ (red closed squares for oils and red open squares for W-G) and *C*_*v*_ (blue closed circles for oils and blue open circles for W-G), plotted as a function of Oh. Red and blue dashed lines are averages of data points. Red solid lines: *C*_*i*_ ≈ 2 for Oh < 0.1 and *C*_*i*_ ≈ Oh^−1^ for Oh > 10. Blue solid lines: *C*_*v*_ ≈ Oh for Oh < 0.1 and *C*_*v*_ ≈ 0.5 for Oh > 10.

We measured the radii of air films with time, taking from the maximum lengths of the entrained air films on the X-ray images, as plotted in Fig. 4(a). Importantly, the retraction rate *R*(*t*)/*R*_0_ follows the inertial exponential decay for liquids with Oh < 0.1. For liquids with Oh > 0.1, however, the retraction rate significantly deviates from the exponential decay. The best-fit values of the proportional coefficients *C*_*i*_ and *C*_*v*_ for each liquid are plotted in Fig. 4(b). At Oh < 0.1, we find that *C*_*i*_ is almost invariant (1.3 ~ 1.8) when *C*_*v*_ linearly increases with Oh, indicating the inertial retraction. In contrast, at Oh > 10, *C*_*v*_ saturates to ~0.5 when *C*_*i*_ is inversely proportional to Oh, indicating the viscous retraction. The comparable values of *C*_*i*_ and *C*_*v*_ at intermediate ranges (0.1 < Oh < 10) indicate that the inertia and the viscosity influence the retraction dynamics. From Eqs. (1) and (2), the specific condition for *C*_*i*_ = *C*_*v*_ (or *τ*_*i*_ = *τ*_*v*_) is expected at Oh $=\sqrt{4/3}\approx 1.15$, as consistent with Fig. 4(b).

Interestingly, a slight difference in *C*_*i*_ for oils (*C*_*i*_ ~ 1.8) and aqueous solutions (*C*_*i*_ ~ 1.3) was found at the inertial regime (Oh < 0.1). This can be explained by the hydrodynamic instability by large surface tension as in aqueous liquids^20^. Necklace rims are formed in aqueous liquids in Fig. 3, while smooth rims are formed in alkane liquids in Fig. 2. The necklace rim is responsible for the formation of tiny bubbles (dashed circles in Fig. 3) attributed to the capillary instability along the rim^17,20,43^.

### Phase diagram

**Figure 5:**
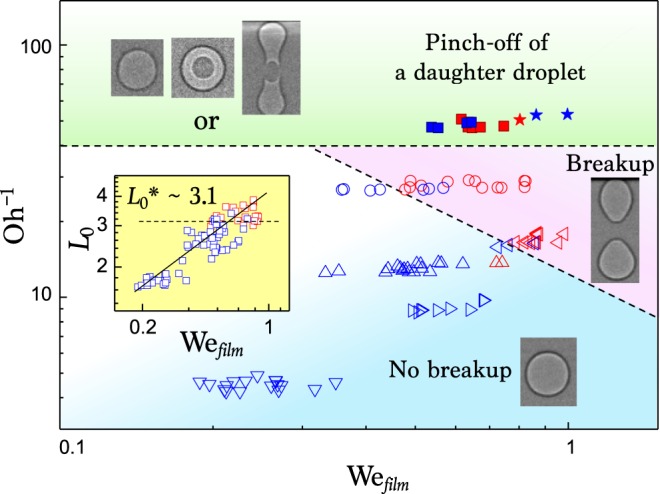
Phase diagram for the bubble evolution with respect to We_*film*_ and Oh^−1^. The shape of symbols is analogue to that in Fig. 4(a). Blue open symbols: the simple retraction into one bubble; red open symbols: the breakup into a double bubble; closed symbols: pinch-off of a daughter droplet; blue closed symbols: single or an antibubble; red closed symbols: a double bubble. Inset: Aspect ratio *L*_*o*_ of a bubble, measured as a function of We_*film*_ for liquids. The solid line is the best fitting of allometric scaling: *y* = *αx*^*β*^.

The evolution of the entrained air is affected by the interaction among inertia, capillarity, and viscosity. As plotted in Fig. 5, we obtain a phase diagram for the final fate of the air as functions of the inverse Ohnesorge number Oh^−1^ and the Weber number for the air film as defined as We_*film*_ = *ρν*^2^*δ*/*γ* which is the ratio of kinetic energy (inertia) to surface tension, where the retraction velocity *ν* is taken from the measured retraction speed $\max (-dR/dt)$^38^. In fact, *ρ* and *γ* were controlled in Table 1 and *ν* and *δ* were measured experimentally in the X-ray imaging. Remarkably, there are three distinct types of air evolution. A simple retraction to one bubble is found at low Oh^−1^ and low We_*film*_ (blue region) and a split of one into two bubbles is found at low Oh^−1^ and high We_*film*_ (red region). At high Oh^−1^ (green region), a daughter droplet is robustly formed but all three cases of one and two bubbles, or a shell bubble are possible.

The bubble breakup can be explained with respect to the competition among inertia, capillarity, and viscosity on the bubble columns vertically elongated caused by the inertia of retraction, as seen in Figs. 2 and 3. This inertia of retraction, or retraction speed, is proportional to the impact speed of liquid drop because the initial thickness of air film as well as the size of trapped air bubble become smaller with the impact speed^12^. Thus, as seen in the inset of Fig. 5, the aspect ratio *L*_*o*_ of the bubbles, measured at their maximum elongation (for example, at *t* = 96 *μ*s in Fig. 2(c)), increases with the kinetic energy of impacting liquid drop as *L*_*o*_ ~ We${}_{film}^{\beta }$, where the best-fit exponent *β* is measured as ~0.486 ± 0.037. We find that the breakup usually occurs at high aspect ratios larger than a critical value, ${L}_{o}^{* } \sim 3.1$ in our data. The critical aspect ratio for typical breakup of fluid columns increases with fluid viscosity^44–47^. Thus, it is conceivable that the breakup would be suppressed at high liquid viscosity, i.e. high Oh. Generally, ${L}_{o}^{* }$ for the breakup is predicted to be proportional to Oh^1/2^ based on linear instability theory^45,46^. From the relations of *L*_*o*_ ~  We${}_{film}^{\beta }$ and ${L}_{o}^{* } \sim$ Oh^1/2^, the dependence of the critical We${}_{film}^{* }$ on Oh can be rationalized as We${}_{film}^{* }$ ~  Oh^(1/2*β*)^. This explains the boundary between breakup and no breakup in the phase diagram (Fig. 5), which is well matched with experimental data.

**Figure 6:**
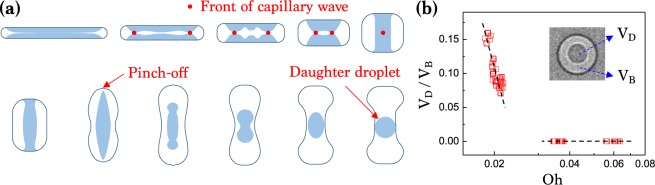
Formation of a daughter droplet. (**a**) Illustration of pinch-off of a daughter droplet by capillary waves. (**b**) The ratio of the daughter droplet volume (*V*_*D*_) to the air bubble volume (*V*_*B*_) measured as a function of Oh.

The formation of daughter droplets at small Oh (< ~ 0.025) in the green region of Fig. 5 is because of the capillary waves on the air film, similar to the case of drop impact on solid surfaces^13,48^. At the moment of entrapment of the air disc by drop impact on surfaces, capillary waves are generated at the disc edge and travel into the center. As the waves converge, their amplitudes grow and cause the contact of liquids from upside and downside, forming the air film into a toroidal shape. Then pinch-off of a daughter droplet occurs inside the bubble. This process is well illustrated in Fig. 6(a). The capillary waves are balanced with the viscous dissipation: the critical Oh values are 0.026 or (Oh*)^−1^ = 38.46 for droplet pinch-off^49^ and 0.052 or (Oh*)^−1^ = 19.23 for bubble pinch-off  ^4^. Therefore, the daughter droplet can be formed for low-viscosity liquids with Oh^−1^ > 40 (Oh < 0.025) in Fig. 5. Additionally, decreasing Oh at Oh < 0.03 would increase the daughter droplet size, as seen in the ratio of the daughter droplet volume (*V*_*D*_) to the bubble volume (*V*_*B*_) measured as a function of Oh in Fig. 6(b). The pinch-off driven formation of the daughter droplet at Oh^−1^ > 40 or Oh < 0.025 is consistent between the theory^4,49^ and the experiment in Figs. 5 and 6(b).

**Figure 7:**
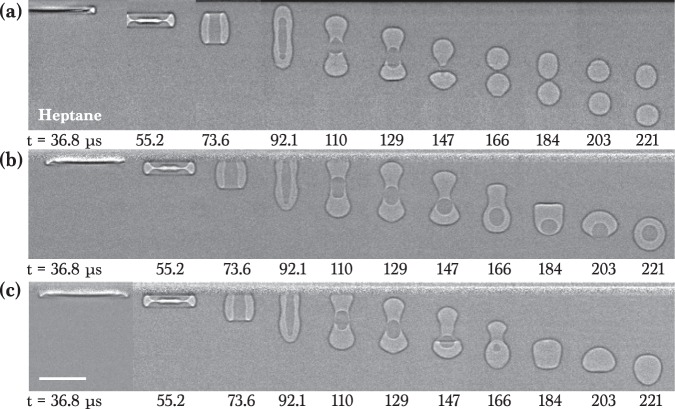
Sequential X-ray images of evolution of the entrapped bubble for the heptane. (**a**) The axisymmetric coalescence of the daughter droplet and the surrounding liquid splits the bubble into a double bubble. (**b**) No coalescence results in an antibubble comprising the daughter droplet inside. (**c**) One-sided coalescence of the daughter droplet with the surrounding liquid results in one bubble. The scale bar is 100 *μ*m long.

**Figure 8:**
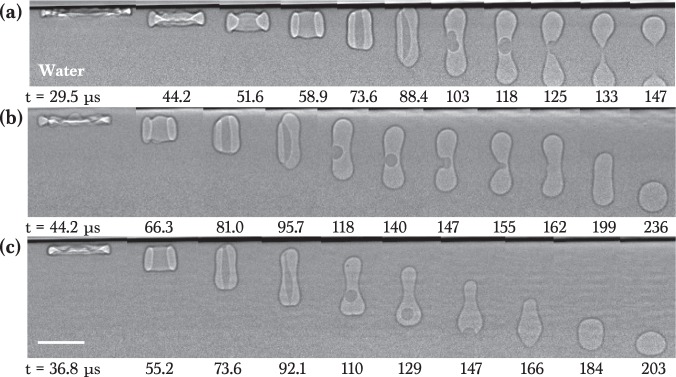
Sequential X-ray images of evolution of the entrapped bubble for water. (**a**) The axisymmetric coalescence forms the bubble split into two bubbles. (**b**) The one-sided coalescence results in one bubble. (**c**) The bottom-sided coalescence of the daughter droplet in a peanut-like bubble also results in one bubble. The scale bar is 100 *μ*m long.

The interaction of a daughter droplet with the surrounding liquid plays a crucial role in determining the final fate of the bubble. For the heptane, the daughter droplet coalesces axisymmetrically with the surrounding liquid and splits the bubble into a double bubble (Fig. 7(a)) or does not coalesce and instead remains inside the shell bubble, forming an antibubble (Fig. 7(b)). In rare cases, the daughter droplet forms one-sided coalescence with the surrounding liquid, resulting in one bubble (Fig. 7(c)). For water, the daughter droplet forms total coalescence (Fig. 8(a)) or one-sided coalescence at the equator of the vertically elongated bubble (Fig. 8(b)). In some events, the daughter droplet can coalesce with the surrounding liquid at the bottom of the bubble, resulting in one bubble as well (Fig. 8(c)). The coalescence is a random process and may not always happen in the center^41^. For water, there was no event of shell bubble formation in our experiments.

**Figure 9:**
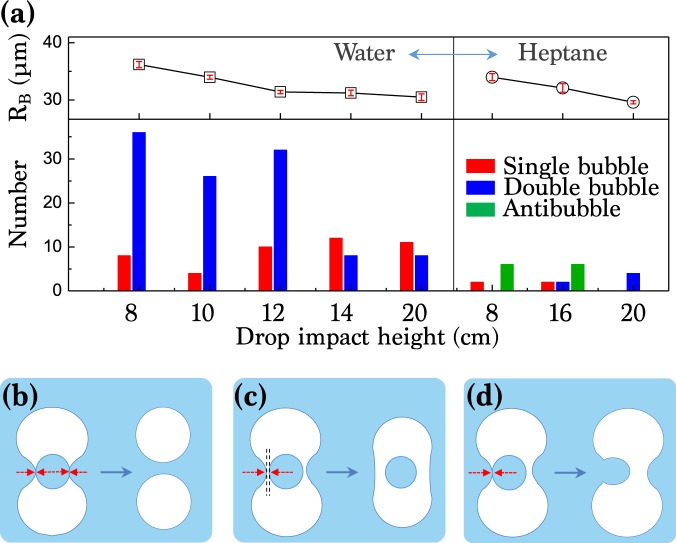
The fate of entrapped air. (**a**) (bottom) Probability density of the final fate of the entrapped bubbles for water (left) and the heptane (right) at different impact heights. (top) Average radius of the bubbles of water (left) and the heptane (right) at different impact heights. Schematic illustrations for (**b**) bubble breakup by total coalescence (a double bubble), (**c**) shell bubble formation (an antibubble), and (**d**) one-sided coalescence (one bubble).

The frequency of all the cases of single, double, or anti- bubbles at various experimental conditions are plotted in Fig. 9(a). The radius of the final spherical bubble *R*_*B*_ is the average radius of the final single, double, or anti- bubbles, taken from the final bubble images. In a view of typical hydrodynamics, the air layer between surrounding liquid and the daughter droplet becomes very thin (< 1 *μ*m) after the pinch-off. Thus, the layer is easily broken in water because of its high disjoining pressure, resulting in the split of the bubble into two, as illustrated in Fig. 9(b). Conversely, the thin air layer is not easily broken in oil because of its small surface tension, thus playing as a lubricating layer between the daughter droplet and surrounding oil, as illustrated in Fig. 9(c). Additionally, hydrodynamic instability may induce one-sided coalescence of the daughter droplet, eventually forming one bubble, as illustrated in Fig. 9(d). One interesting point in Fig. 9(a) is that for water, the events of no-split for one bubble are more frequently observed at high impact heights. This can be explained by the decrease in the bubble size with impact height, as shown in the top panel of Fig. 9(a). The increase in Oh by the decreased bubble size results in the decrease of the daughter droplet size, reducing the coalescence probability.

## Discussion

**Figure 10:**
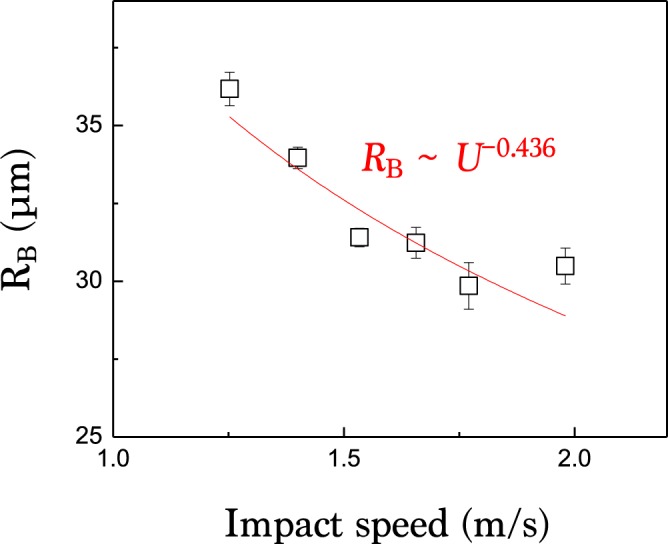
Average bubble radius as a function of drop impact speed for water. The red line is fitted based on a power-law: *R*_*B*_ ~ *U*^−0.436^.

The speed of impacting drop can significantly affect the initial dynamics of air. When the speed is low as *U* < 0.5 m/s, rupture occurs at numerous locations simultaneously, entrapping a multitude of tiny bubbles, known as the Mesler entrainment^18,50^. For *U* > 0.5 m/s, the air layer under the drop ruptures along an azimuthal ring and a disc-shaped air film is stably entrapped^18,20^. Especially, for *U* = 1.0 ~ 2.5 m/s, as applied here, the evolution dynamics of the air films is hardly affected by the impacting speed, except for their size dependency on the speed^19^, as fitted by *R*_*B*_ ~ *U*^−0.436^ in Fig. 10. This relation estimates *R*_*B*_ ~ 70 *μ*m at *U* ~ 0.25 m/s, as consistent with the previous measurement of *R*_*B*_ ~ 60*μ*m^51^ for an ethanol droplet of 0.9 mm radius at *U* ~ 0.25 m/s.

Here, the Weber number of the impacting drop was markedly similar between this study for drop impact on liquid pools (We_*drop*_ ~  50–210) and previous studies for drop impact on solid substrates (55–70^13^ or 70–900^48^), but the below boundary condition was markedly different. This study clearly reveals the variety of air evolution dynamics. At very high impact speeds, collapsing of the impact crater would be a main mechanism for the bubble entrainment^16^, generally known as regular bubble entrainment. Also, unusual phenomena such as the cascade entrapment of bubble-rings^21^, and the formation of vortex streets also can occur^52^. By expanding the experimental conditions, one may be able to explore such singular hydrodynamic behavior in drop impact on liquid using novel techniques such as ultrafast X-ray phase-contrast imaging.

Finally, we did not consider the effect of variation of air properties such as air temperature and viscosity. The change in air temperature can modify the properties of liquid, in particular liquid viscosity (generally, the viscosity becomes smaller at a higher temperature), and thus can shift the boundaries between regions in the phase diagram. The change in air viscosity significantly affects the whole dynamics of drop impact^53^ and thus it would be crucial to investigate the effect of air viscosity on air entrapment during drop impact on a liquid pool. Further studies should be expanded to understand the oblique impact of droplets on deep liquid pools^54–56^, the impact of viscoelastic or viscous droplets^57–59^, and the impact-induced fabrication^60,61^, with more facilitating of numerical^62^, theoretical^63^, and experimental approaches^64,65^.

## Conclusion

In summary, the novel ultrafast X-ray phase-contrast imaging is useful for us to explore the dynamics of the air entrainment during drop impact on a liquid pool. We elaborated two retraction mechanisms of the entrapped air film, inertial and viscous retractions, which strongly depend on the liquid viscosity. We found two crucial dynamic singularities after retraction: (i) the breakup of the bubble, mostly characterized with the bubble geometry and the liquid property, and (ii) the pinch-off of the daughter droplet (at Oh < 0.025) and its effect on the final fate of the bubble, which have been unachievable using conventional imaging techniques. Finally, predicting the morphological evolution of the entrained air would be assessable via the phase diagram with respect to hydrodynamic conditions.

## Supplementary information

Supplementary Information.

Supplementary Information2.

Supplementary Information3.

Supplementary Information4.

Supplementary Information5.

Supplementary Information6.

Supplementary Information7.

Supplementary Information8.
