# Supplementary material for: Air evolution during drop impact on liquid pool
Source: Sci Rep. 2020 Apr 1;10:5790. doi: 10.1038/s41598-020-62705-5 (PMC7113293; doi:10.1038/s41598-020-62705-5)
Supplement: Supplementary file 8 — Supplementary Information8. [file 41598_2020_62705_MOESM8_ESM.pdf]

Supplementary Information

Air evolution during drop impact on liquid pool

Ji San Lee[1], Byung Mook Weon[2,3,4*], Su Ji Park[1], Ji Tae Kim[1], Jaeyeon Pyo[1], Kamel Fezzaa[5], and Jung Ho Je[1,†]

Affiliations:

[1] X-ray Imaging Center, Department of Materials Science and Engineering, Pohang University of Science and Technology, 77 Cheongam-Ro, Nam-Gu, Pohang 37673, South Korea

[2] Soft Matter Physics Laboratory, School of Advanced Materials Science and Engineering, SKKU Advanced Institute of Nanotechnology (SAINT), Sungkyunkwan University, Suwon 16419, South Korea

[3] Research Center for Advanced Materials Technology, Sungkyunkwan University, Suwon 16419, South Korea

[4] Department of Biomedical Engineering, Johns Hopkins University, Baltimore, Maryland 21218, USA

[5] X-ray Science Division, Advanced Photon Source, Argonne National Laboratory, 9700 South Cass Avenue, Argonne, Illinois 60439, USA

* Correspondence and requests for materials should be addressed to B.M.W. (bmweon@skku.edu) or J.H.J. (jhje@postech.ac.kr).

Supplementary Video Legends:

Movie S1. Clear visualization for bubble entrapment during impact of a pentadecane drop obtained by ultrafast X-ray imaging, which is the original movie of sequential images in Fig. 2(a).

Movie S2. Clear visualization for bubble entrapment during impact of a dodecane drop obtained by ultrafast X-ray imaging, which is the original movie of sequential images in Fig. 2(b).

Movie S3. Clear visualization for bubble entrapment during impact of a heptane drop obtained by ultrafast X-ray imaging, which is the original movie of sequential images in Fig. 2(c).

Movie S4. Clear visualization for bubble entrapment during impact of a $W_{0.4}G_{0.6}$ drop obtained by ultrafast X-ray imaging, which is the original movie of sequential images in Fig. 3(a).

Movie S5. Clear visualization for bubble entrapment during impact of a $W_{0.6}G_{0.4}$ drop obtained by ultrafast X-ray imaging, which is the original movie of sequential images in Fig. 3(b).

Movie S6. Clear visualization for bubble entrapment during impact of a $W_{0.8}G_{0.2}$ drop obtained by ultrafast X-ray imaging, which is the original movie of sequential images in Fig. 3(c).

Movie S7. Clear visualization for bubble entrapment during impact of a water drop obtained by ultrafast X-ray imaging, which is the original movie of sequential images in Fig. 3(d).
